# Down syndrome: Prevalence and distribution of congenital heart disease in Brazil: Síndrome de Down: prevalência e distribuição de cardiopatia congênita no Brasil

**DOI:** 10.1590/1516-3180.2015.00710108

**Published:** 2015-12-08

**Authors:** Beatriz Elizabeth Bagatin Veleda Bermudez, Sandra Lira Medeiros, Mariane Bagatin Bermudez, Iolanda Maria Novadzki, Neiva Isabel Rodrigues Magdalena

**Affiliations:** I MD, MSc. Specialization Student on Down Syndrome at Centro de Estudos e Pesquisas Clínicas de São Paulo; Doctoral Student in Postgraduate Program on Children's and Adolescents' Health, Universidade Federal do Paraná, Curitiba, Paraná, Brazil.; II MD. Specialist in Pediatric Cardiology, Department of Pediatrics, Universidade Federal do Paraná, Curitiba, Paraná, Brazil.; III MD. Psychiatry Resident, Universidade Federal de Ciências da Saúde de Porto Alegre, Porto Alegre, Rio Grande do Sul, Brazil.; IV MD, MSc. Specialist in Pediatrics and Adolescent Medicine, Down Syndrome Outpatient Clinic, Universidade Federal do Paraná, Curitiba, Paraná, Brazil.; V MD, PhD. Professor, Department of Pediatrics, Universidade Federal do Paraná, Curitiba, Paraná, Brazil.

**Keywords:** Heart defects, congenital, Down syndrome, Hypertension, pulmonary, Echocardiography, Cardiac surgical procedures

## Abstract

**CONTEXT AND OBJECTIVE:**

Down syndrome is the most common genetic disorder, affecting 1/700 live births. Among the clinical findings, one constant concern is the high prevalence of congenital heart disease. The objective of this study was to determine the prevalence and profile of congenital heart disease among patients attended at a Down syndrome outpatient clinic in southern Brazil between 2005 and 2013.

**DESIGN AND SETTING:**

: Cross-sectional study conducted in a referral center.

**METHODS:**

: Data were retrospectively gathered from the medical files of 1,207 patients with Down syndrome, among whom 604 (50.0%) had been diagnosed with congenital heart disease. These data were subjected to descriptive analysis using the Statistica software.

**RESULTS:**

: Among the 604 patients with congenital heart disease, 338 (55.8%) were male and 269 (44.5%) were female. The most common heart diseases were atrial septal defect in 254 patients (42.1%); total atrioventricular septal defect in 91 (15.1%); atrial septal defect and ventricular septal defect in 88 (14.6%); ventricular septal defect in 77 (12.7%); patent ductus arteriosus in 40 (6.6%); patent foramen ovale in 34 (5.6%) patients; tetralogy of Fallot in 12 (2%); and other diseases in 8 (1.3%). Pulmonary hypertension was present in 57 (9.4%). Out of the total, 150 patients (24.8%) underwent cardiac surgery.

**CONCLUSION:**

: The high prevalence of congenital heart disease among the patients at the Down syndrome outpatient clinic (50%) was similar to findings from other studies and justifies investigation during the neonatal period, so as to decrease mortality and morbidity.

## RESUMO

**CONTEXTO E OBJETIVO:**

: A síndrome de Down é a anomalia genética mais comum e afeta 1/700 nascidos vivos. Entre os achados clínicos, uma preocupação constante é a alta prevalência de cardiopatia congênita e o objetivo do presente estudo é determinar a prevalência e o perfil de cardiopatia congênita em pacientes atendidos em um ambulatório de síndrome de Down no período de 2005 a 2013 no sul do Brasil.

**TIPO DE ESTUDO E LOCAL:**

: Estudo transversal conduzido em um centro de referência.

**MÉTODOS:**

: Foi realizada coleta retrospectiva de dados de 1.207 prontuários de pacientes com síndrome de Down, com 604 (50,0%) diagnosticados com cardiopatia congênita. Os dados foram submetidos a análise descritiva, utilizando-se o programa Statistica.

**RESULTADOS:**

: Entre os 604 pacientes com cardiopatia congênita, 338 (55,8%) eram do sexo masculino e 269 (44,5%) do feminino. A cardiopatia mais comum foi comunicação interatrial, observada em 254 (42,1%) pacientes, defeito de septo atrioventricular total, em 91 (15,1%), comunicação interatrial em conjunto com comunicação interventricular, em 88 (14,6%), comunicação interventricular, em 77 (12,7%), persistência do canal arterial, em 40 (6,6%), forame oval patente, em 34 (5,6%), tetralogia de Fallot, em 12 (2%) e outras cardiopatias observadas em oito (1,3%) pacientes. Hipertensão pulmonar esteve presente em 57 (9,4%). Do total, 150 (24,8%) pacientes foram submetidos a cirurgia cardíaca.

**CONCLUSÃO:**

: A elevada prevalência das cardiopatias congênitas nos pacientes do ambulatório da síndrome de Down, de 50%, semelhante à encontrada em outros estudos, justifica a investigação no período neonatal, com diminuição da mortalidade e morbidade.

## INTRODUCTION

Down syndrome is the most common genetic disorder and it is characterized by 95% regular trisomy 21 and 5% in the form of translocation and mosaic.^1^ Because of the presence of extra genetic material from chromosome 21, children with Down syndrome have multiple malformations, medical conditions and cognitive impairment.^1^ ^2^ Congenital heart defect is an associated condition in 40% to 60% of Down syndrome patients, but clinical and surgical treatment has increased the survival of these patients over recent decades: 25 years in 1983 to 49 in 1997 and 60 in 2007.^3^

The prevalence of serious infections such as pneumonia and sepsis is high (70% and 85% respectively) among children with Down syndrome and congenital heart disease between the ages of six and 48 months.^4^ Clinical follow-up and periodic vaccination, including use of the antibody against respiratory syncytial virus, is therefore important.^4^

## OBJECTIVE

The objective of the present study was to determine the prevalence and profile of congenital heart disease among patients treated at a Down syndrome clinic in Southern Brazil between 2005 and 2013.

## METHODS

This study was approved by the Research Ethics Committee of the Clinical Hospital of the Federal University of Paraná (Brazil Platform No. 04542712.3.0000.0096). It was conducted in a public referral center for people with Down syndrome that was created in 1997 with the purpose of ensuring full health care for these people, with an interdisciplinary team and family support.

This was a retrospective, observational, descriptive and cross-sectional study using data gathered from 1207 patients' medical records at the Down syndrome clinic covering the period 2005-2013. These patients comprised 667 males (55.3%) and 540 females (44.7%), with a median age at first visit of 2.0 months, ranging from 0.0 to 11.0 months (95% CI: 0-9).

All the patients underwent more than one echocardiography examination: one brought to the first visit and at least one other, requested at this service for follow-ups.

Pulmonary hypertension was defined as mean pulmonary artery pressure greater than 25 mmHg at rest, with no evidence of left atrial hypertension (with a pulmonary capillary wedge pressure < 15 mmHg).

The data were analyzed using descriptive statistics, by means of the Statistica software.

## RESULTS

The mothers' mean age at the time of their child's birth was 32.1 ± 8.6 years, ranging from 12 to 49 years (95% CI: 31.6 to 32.6). Among the mothers of the Down syndrome patients, 18.6% were primiparous and 81.4% were multiparous.

Congenital heart defects were present in 604 patients (50%) with Down syndrome and among these, 338 (55.8%) were male and 269 (44.5%) were female. The most common heart disease diagnosed was atrial septal defect in 254 patients (42.1%), followed by complete atrioventricular septal defect in 91 (15.1%); atrial septal defect and ventricular septal defect in 88 (14.6%); ventricular septal defect in 77 (12.7%); patent ductus arteriosus in 40 (6.6%); patent foramen ovale in 34 (5.6%); tetralogy of Fallot in 12 (2%); and other heart diseases in 8 (1.3%), as shown in Table 1. Pulmonary hypertension was present in 57 cases (9.4%). Out of the total, 150 patients (24.8%) underwent cardiac surgery. Only 272 patients (45%) with congenital heart disease were brought to our service before the age of three months; 376 (62%) were brought before the age of six months and 424 (70%) before the age of one year.

**Table 1.** Prevalence of congenital heart disease among 604 children with Down syndrome

Disease type	Number of patients	Percentage
Atrial septal defect	254	42.1
Atrioventricular septal defect	91	15.1
Atrial septum defect and ventricular septum defect	88	14.6
Ventricular septal defect	77	12.7
Patent ductus arteriosus	40	6.6
Patent foramen ovale	34	5.6
Tetralogy of Fallot	12	2.0
Other	8	1.3
Total	604	100

## DISCUSSION

The prevalence of congenital heart disease was similar to the findings from other studies, which have been between 44% and 62%.^3^ ^5^ ^17^ The types of heart defect may vary according to geographic region. In this study, the most common type was atrial septal defect, which was similar to the findings from other Brazilian,^1^ ^4^ ^8^ South Korean^5^ and Libyan^9^ studies, but different from others in the United States,^6^ ^17^ France,^15^ ^16^ Turkey^11^ and Sweden,^15^ where atrioventricular septal defect was more frequent. The most common single cardiac lesion was ventricular septal defect in China (in approximately 40% of Down syndrome children)^12^ and patent ductus arteriosus in Guatemala^13^ and Mexico.^14^ Nonetheless, in United States, Freeman et al. found that the most common type of congenital heart disease was atrioventricular septal defect (47%), in the Atlanta Down Syndrome Project in 1998^5^ and, ten years later, the rates for ventricular septal defect (19.2%), atrial septal defect (18.6%) and atrioventricular septal defect (17.2%) were similar in a report from the National Down Syndrome Project.^6^

Among 138 patients at a pediatric cardiology clinic, Down syndrome was more prevalent among females (56.1%). Females also had higher prevalence of congenital heart disease (81.2%) and a higher rate of association with pulmonary hypertension (37.2% versus 9.4%).^1^ The most common heart disease was atrial septal defect (51.8% versus 42.1%), followed by complete atrioventricular septal defect (45.5% versus 15.1%).^1^ The lower prevalence of complete atrioventricular septal defect in our sample explains our lower association with pulmonary hypertension. Our results were different from those of another survey conducted in the same clinic.^4^ The prevalence of congenital heart disease was higher when a smaller age group (6-48 months) was considered, amounting to 70.1% of 127 children, among whom 37.7% required surgical correction.^4^ However, atrial septal defect was the most common type of heart disease in both surveys, with prevalence ranging from 40.1% to 42.1%. This differed from the other Brazilian,^8^ Libyan^9^ and Korean^5^ studies, in which the prevalence ranged from 23% to 57.1%.

Although our clinic is a referral center, only 42% of the Down syndrome patients with congenital heart disease were brought for their first medical visit before the age of three months, while 62% were brought within their first six months of life. Many of them had not been investigated for the possibility of heart disease, but the search rate was better than among pediatric cardiology clinic patients.^1^ This is worrying, since early diagnosis coupled with effective surgical treatment is the factor mainly responsible for decreasing the morbidity and mortality rates in this population.^1^

The present study has limitations, because of its use of noninvasive assessment, rather than cardiac catheterization, for establishing the diagnosis of pulmonary hypertension; and also because of the low percentage of referrals before the age of three months. This highlights the need for better ranking of Down syndrome patients, in the context of congenital heart disease, because of the high frequency and progression of pulmonary hypertension, which hinders the possibility of successful heart surgery. On the other hand, this study is valid because of its sample size.

## CONCLUSION

The high prevalence of congenital heart disease among Down syndrome patients (50%) justifies its investigation during the neonatal period, with the aims of decreasing the mortality and morbidity rates, having fewer visits to clinics or hospitalization and ensuring better operating conditions, if this becomes necessary, with less suffering for patients and their families, lower costs and improvement of overall health, wellness and development.
